# Adult Neurogenesis under Control of the Circadian System

**DOI:** 10.3390/cells11050764

**Published:** 2022-02-22

**Authors:** Amira A. H. Ali, Charlotte von Gall

**Affiliations:** Institute of Anatomy II, Medical Faculty, Heinrich Heine University, Moorenstrasse 5, 40225 Dusseldorf, Germany; amira.ali@med.uni-duesseldorf.de

**Keywords:** circadian system, suprachiasmatic nucleus, molecular clockwork, clock genes, adult neurogenesis, hippocampus, redox state, reactive oxygen species, melatonin, corticosterone, light, neurodegeneration

## Abstract

The mammalian circadian system is a hierarchically organized system, which controls a 24-h periodicity in a wide variety of body and brain functions and physiological processes. There is increasing evidence that the circadian system modulates the complex multistep process of adult neurogenesis, which is crucial for brain plasticity. This modulatory effect may be exercised via rhythmic systemic factors including neurotransmitters, hormones and neurotrophic factors as well as rhythmic behavior and physiology or via intrinsic factors within the neural progenitor cells such as the redox state and clock genes/molecular clockwork. In this review, we discuss the role of the circadian system for adult neurogenesis at both the systemic and the cellular levels. Better understanding of the role of the circadian system in modulation of adult neurogenesis can help develop new treatment strategies to improve the cognitive deterioration associated with chronodisruption due to detrimental light regimes or neurodegenerative diseases.

## 1. The Mammalian Circadian System

**Figure 1:**
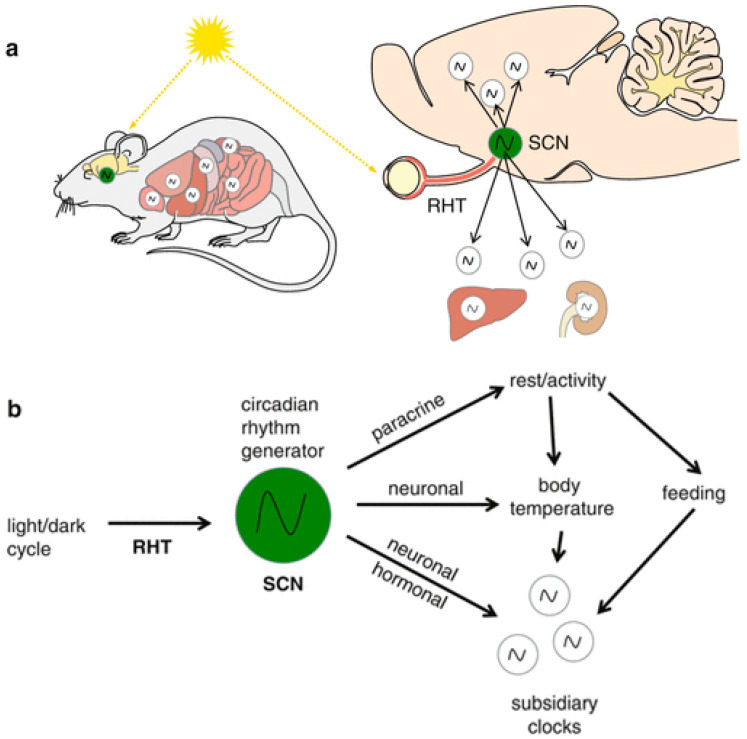
The mammalian circadian system: (**a**) The circadian rhythm generator in the suprachiasmatic nucleus (SCN) is entrained to light information conveyed to the brain from the retina via the retinohypothalamic tract (RHT). (**b**) The time information is then transmitted from the SCN to subsidiary circadian oscillators in the brain and the body via neuronal networks and hormones to regulate rhythmic brain and body functions [Reprinted with permission from ref. [2]. Copyright 2013 Springer Nature].

The circadian clock has developed very early during evolution to enable the organisms to anticipate daily rhythms in the environment such as the light/dark cycle. In mammals, the circadian clock is a hierarchically organized multicomponent internal time-keeping system that synchronizes rhythms in physiology, metabolism and behavior to the 24 h solar day (Circa: about, dies: a day) [reviewed by [1]]. The circadian rhythm generator, residing in the hypothalamic suprachiasmatic nucleus (SCN), is entrained to rhythmic external cues such as the environmental changes in the light/dark cycle. Light is considered the most pronounced time giver (zeitgeber) that synchronizes internal and external time. The SCN receives time information and, in turn, coordinates the subordinate clocks in the brain and the periphery. This coordination occurs via direct and indirect neuronal connections, body temperature as well as hormones and results in modulation of the rhythmic behavior and body functions according to anticipated time-of-day-changing demands [2] (Figure 1).

**Figure 2:**
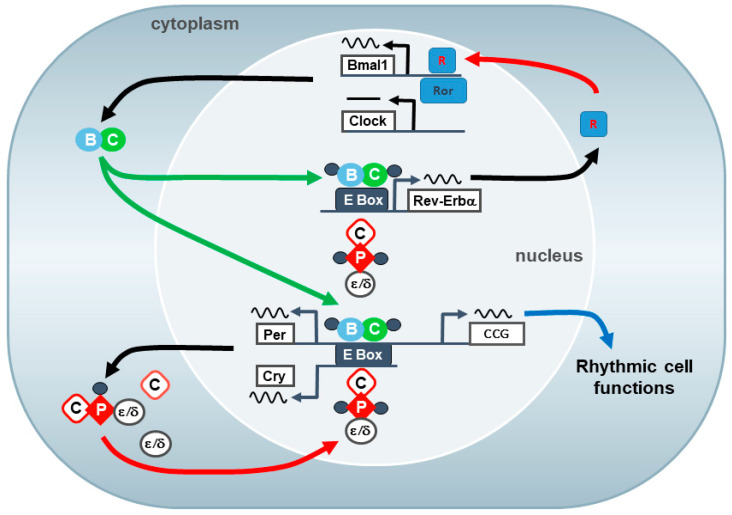
The molecular clockwork: The molecular clockwork consists of autoregulatory transcriptional/translational feedback loops of clock genes that produce a 24-h (circadian) rhythm in gene expression and cell function. The core clock loop comprises the two transcription factors CLOCK (C) and BMAL1 (B) in addition to two families of gene expression inhibitors: the PERs and the CRYs. The CLOCK and BMAL1 complex activates the transcription of the *Period* (*Per*) and *cryptochrome* (*Cry*) genes and clock controlled genes (*ccg*) via E-box (like) enhancer elements. The Per (P) and Cry proteins (C) form a repressor complex that also comprises casein kinase 1ε or δ (ε, δ). After translocation into the nucleus, the repressor complex inhibits CLOCK:BMAL1-mediated transcription. A new cycle starts after ubiquitination and proteasomal degradation of the repressor complex. An accessory feedback loop, including the orphan nuclear receptors REV-ERBα and RORα, modulate the core loop via binding to ROR enhancer elements and regulation of the Bmal1 gene [Reprinted with permission from ref. [2]. Copyright 2013 Springer Nature, after [4]].

Almost each cell within the body possesses cell autonomous timekeeping properties, which are orchestrated by the SCN [3]. The cell intrinsic molecular clockwork that drives rhythmic cell function is composed of two interlocking transcriptional–translational feedback loops that produce a 24-h (circadian) rhythm in gene expression. The core clock components are the two transcription factors CLOCK and BMAL1 in addition to two families of gene expression inhibitors: the PER (PER1 and PER2) and the CRY (CRY1 and CRY2) [4]. At the beginning of the cycle, the positive components, the transcription factors CLOCK and BMAL1, heterodimerize and bind to E-box elements of *Per* and *Cry* genes leading to enhancing their transcription. Thereafter, the *Per* and *Cry* are translated into their respective proteins (PER and CRY) [1]. Late in the cycle, the negative components, the PERs and CRY, form together with casein kinases a negative regulatory complex that translocate from the cytoplasm to the nucleus and bind to *Clock* and *Bmal1* promoter leading to inhibition of *Clock/Bmal1* transcription. When PER and CRY proteins undergo degradation, this inhibitory effect is released and *Clock/Bmal1* mediated transcription is resumed [4]. A secondary feedback loop comprising the transcriptional activator retinoid-related orphan receptor (ROR) and the transcriptional repressor orphan nuclear receptor REV-ERBα modulates the rhythmic transcription of *Bmal1*. In turn, CLOCK:BMAL1 regulate rhythmic *Rev-Erbα* transcription. This secondary feedback loop provides an additional stabilization mechanism for the molecular clockwork [5] (Figure 2). The circadian phase of rhythmic clock gene expression may vary among different brain structures and organs.

It is worth mentioning that E-box elements exist in the promoters of a wide variety of genes encoding for key cell function modulators, which are known as “clock-controlled genes” (*ccg*). Therefore, the molecular clockwork regulates rhythmic cell function in addition to their role in time keeping [6]. The role of clock genes and the molecular clockwork in adult neurogenesis will be later discussed in detail.

Entrainment of the molecular clockwork in the SCN neurons to the environmental light/dark cycle is achieved via retinal afferents that project from the eye and light-induced signal transduction [reviewed by [1]]. Interestingly, the molecular clockwork is self-sustained and continues to oscillate in the absence of rhythmic environmental cues, e.g., under constant darkness and even under culture conditions, with a circadian period [7]. Nevertheless, circadian rhythms in isolated SCN neurons show slightly different phases [reviewed in [8]]. Therefore, a tight coupling of SCN neurons is crucial for robust rhythmicity and phase coherence [9]. Within the SCN, intercellular communication via gap junctions (electrical coupling) and synapses (chemical coupling) is critical for conveying input to and output from the SCN and, thus, circadian rhythm precision [10]. We showed recently that the gap junction proteins Cx30 and Cx43 are rhythmically expressed in the SCN and contribute to entrainment under challenging conditions [11].

Anatomically, the SCN is subdivided into the ventrolateral (core) and dorsomedial (shell) regions. The ventrolateral neurons receive photic information from the intrinsically photosensitive melanopsin-positive retinal ganglion cells (ipRGCs) through the retino-hypothalamic tract (RHT) [12]. The axons of the ipRGCs releases glutamate and pituitary adenylate cyclase-activating peptide (PACAP). Both neurotransmitters evoke clock gene expression and stimulate SCN neuronal activity [13]. In addition to photic input, the SCN rhythmic activity is modulated by non-photic information about changes of locomotor activity and arousal resulting in modulation of SCN neuronal activity. The non-photic input is mainly conveyed from the intergeniculate leaflet (IGL) via the geniculo-hypothalamic tract through GABA and neuropeptide Y (NPY) release as well as from the dorsal and median raphe nuclei via serotonin (5-HT) [reviewed by [9]].

Importantly, light information also reaches the hippocampus, one of the main neurogenic niches, via either an SCN-dependent and/or an SCN-independent pathway [14,15], and is capable of inducing different signal transduction pathways thus, affecting the hippocampal function [16,17,18].

The SCN coordinates the peripheral clocks via various outputs, directly via neuronal synaptic connections with other brain areas and indirectly through the autonomic nervous system, regulation of hormones, behavior, e.g., food intake and locomotor activity as well as via body temperature [reviewed by [8]]. SCN neurons send projections to multiple targets in the thalamus and hypothalamus as well as some limbic system structures [reviewed by [9]]. Furthermore, SCN regulates rhythmic hormone release through various pathways: the rhythmically released pineal gland hormone melatonin is controlled via the autonomic nervous system [19], while the rhythms in adrenal gland hormone glucocorticoid is controlled via the hypothalamic-pituitary-adrenal axis (HPA) [20]. Disruption within the circadian system has negative effects on physical and mental health and is associated with various metabolic and neurodegenerative diseases including Parkinson’s, Alzheimer’s and Huntington’s diseases [reviewed by [21]].

## 2. Adult Neurogenesis

The generation of new neurons in the adult brain, known as ‘’adult neurogenesis’’, continues throughout life. Adult neurogenesis is a complex multistep process, consisting of proliferation of neuronal stem/precursor cells (NPCs), migration of neuroblasts, apoptosis/survival during critical periods of development, differentiation into mature neurons, and, finally, functional integration into preexisting neuronal circuitries [reviewed by [22]]. Under normal physiological conditions, adult neurogenesis occurs principally in particular regions of the brain, the “neurogenic niches” including the subgranular zone (SGZ) of the hippocampal dentate gyrus (DG) and the subventricular zone (SVZ) of the lateral ventricles, where the a unique microenvironment provides permissive milieu for various adult neurogenesis stages [reviewed by [23]].

Both niches share common features with small differences. In the SGZ, the radial- glia cell-like NPCs (type 1 cells) proliferate and give rise to intermediate progenitors (type 2a cells), which generate doublecortin (DCX) expressing neuroblasts (type 2b cells). Neuroblasts migrate for a short distance to reach a final position within the DG and give rise to immature neurons (type 3 cells). Only a fraction of the neuroblasts survive and differentiate into granule cells that express the mature neuronal marker NeuN and extend their dendrites within the molecular layer and their axons through the hilus to reach the CA3 region to become integrated into the hippocampal neuronal network [24,25]. While in the SVZ, the activated radial-like NPCs (type B cells) in the wall of the lateral ventricle proliferate and generate transient amplifying cells (type C cells), which in turn give rise to neuroblasts (type A cells). The neuroblasts migrate in chains for a longer distance along the rostral migratory stream (RMS), supported by astrocytic scaffolds and guided by neurotrophic factors and cytokines, into the olfactory bulb (OB) where they detach and differentiate into interneurons that integrate into the granule cell layer (GCL) and the glomerular layer (GL) [reviewed by [25]].

In both neurogenic niches, the highly dynamic process of adult neurogenesis is influenced by several extrinsic and intrinsic factors such as enriched environment [26], social interaction [27,28], physical exercise [reviewed in [29]], aging [30], stress [31], Bmal1-deficiency [32,33], diet [34], several neurotransmitters [35] and hormones [36]. Neuroinflammation can affect adult neurogenesis in both directions: acute inflammation has proneurogenic effects that promote CNS repair, while chronic inflammation results in long lasting damage with antineurogenic effects [37]. The modulatory effect of these factors on the distinct steps of adult neurogenesis is exerted via several signaling pathways including Notch, Hedgehog and Wnt signaling, growth- and neurotrophic-factors, cytokines, transcription factors and epigenetic modifications [23]. Furthermore, region specific regulation of adult neurogenesis has been described [33,38,39,40].

Although most research on adult neurogenesis research focuses on the two major niches in DG and SVZ, many studies have reported that it also occurs in other brain areas including the hypothalamus, amygdala and striatum. However, the stability and functional implication of these ‘’non-classical’’ neurogenic niches are controversial [41,42].

Neurogenesis is a critical player for neural plasticity, homeostasis and maintenance of the central nervous system to keep brain function intact and to compensate for neuronal loss caused by aging or pathological events [34]. The hippocampal newborn neurons are essential for learning and memory [reviewed by [43]]. In particular, spatial and object recognition memory [44], memory consolidation [45] and pattern separation, which allows the formation of distinct non-overlapping memories from similar experiences, are considered DG-dependent functions [46]. In addition, adult hippocampal neurogenesis contributes to stress response and emotion [47,48]. In the OB, the newly born interneurons play a critical role in olfactory discrimination [49].

Although the existence of human adult neurogenesis is widely accepted within the scientific community, there is some debate as to its significance [50,51,52]. Importantly, in both human and rodents, altered adult neurogenesis seems to be a hallmark of the neurodegenerative diseases [53,54]. Therefore, a better understanding of factors that modulate adult neurogenesis may provide potential therapeutic strategies for preventing and/or improving cognitive decline associated with neurodegenerative diseases.

## 3. Interaction of the Circadian System and Adult Neurogenesis

Extrinsic factors such as light have a strong effect on both the circadian system and adult neurogenesis, especially in nocturnal rodents. The circadian system controls rhythms in behavior, physiology, hormone secretion and brain metabolism. Many of these factors modulate adult neurogenesis. At the cellular level, the molecular clockwork and oxidative stress tune adult neurogenesis. In the following, we will discuss these multilevel interactions between the circadian system and adult neurogenesis. In addition, we will highlight the effect of a disrupted circadian system on adult neurogenesis.

### 3.1. Light and Chronodisruption

Light is the strongest cue for the entrainment of circadian rhythms to the external environment [reviewed by [6]]. Importantly, rhythmic light/dark cycles control rhythmic physiology and behavior even in the absence of a functional molecular clockwork given an intact light perception [55,56]. Particularly in nocturnal rodents, light suppresses activity and induces anxiety [57,58]. Light information reaches the hippocampus via distinct SCN-dependent [14,15] and independent pathways [14,59]. There is evidence that light exposure [16,17] and light intensity [60] affect hippocampus-dependent learning and memory, probably via increasing hippocampal active p21-activated kinase 1 (PAK1) and enhancing CA1 long-term potentiation [17]. However, little is known if neurogenesis is improved by rhythmic light/dark conditions in comparison to constant dark conditions in which circadian rhythms persist.

Disruption of circadian rhythms, so called chronodisruption, induced by aging, neurodegenerative diseases or detrimental light regimes has an adverse impact on body and brain [reviewed by [61,62]]. Here, we will discuss the impact of circadian disruption induced by constant light/light at night, and chronic phase shifts, e.g., frequent traveling across time zones. Constant light conditions impair hippocampal neurogenesis as well as cognitive performance [63]. Even exposure to dim light at night is associated with reduced expression of hippocampal neurotrophic factors [64]. These data have high relevance for the aversive effects of light at night, e.g., by the use of electronic devices, on cognitive performance and mental health.

Circadian dysfunction induced by chronic phase shifts significantly affects adult neurogenesis and related brain function such as hippocampus-dependent learning and memory in various species. Importantly, the detrimental effect of chronic jet lag on adult neurogenesis is duration-dependent and only induced by phase advance but not by phase delays, thus direction-specific [65,66]. This is consistent with a higher mortality rate [67] and with a stronger disruptive effect on clock gene expression in the SCN [reviewed by [68]] in chronic phase advance as compared to chronic phase delays. In rats, chronic phase shifts have detrimental effects on hippocampal NPC proliferation and dendritic complexity in immature neurons, as well as on memory and mood-related behaviors [65]. In hamsters, chronic phase shifts reduce proliferation of NPCs and formation/survival of newborn neurons in the hippocampus by >50% and disrupt hippocampus-dependent learning and memory [69]. Importantly, chronic phase shift is associated with an increase in serum cortisol levels [69]. Interestingly, while chronic phase shift impacts NPC proliferation in part by the activation of the HPA axis (see below), the reduced formation/survival of newborn neurons seems to be independent on glucocorticoids [69]. In C57BL/6 mice, chronic phase shifts results in a reduction in hippocampal NPC proliferation by 24% and impaired hippocampus-dependent cognitive performance [70]. Importantly, as C57BL/6 mice are melatonin-deficient (see below), the effect of the chronic phase shift on adult neurogenesis and cognitive performance is, at least partly, independent on melatonin. Of note, administration of melatonin alleviates the detrimental effect of chronic jet lag on NPC proliferation and cognitive performance [70] consistent with its pro-neurogenic capacity.

### 3.2. Hormones

Glucocorticoids and melatonin are two major rhythmic endocrine signals of the circadian system acting as synchronizers for subsidiary clocks in the brain and the periphery. They will be reviewed below as they are remarkably involved in the modulation of adult neurogenesis.

#### 3.2.1. Glucocorticoids

The HPA axis comprises parvocellular endocrine neurons in the hypothalamic paraventricular nucleus (PVN) that secrete corticotropin-releasing hormone (CRH) leading to stimulation of the anterior pituitary and release of the adrenocorticotropic hormone (ACTH). ACTH stimulates the adrenal cortex leading to glucocorticoids secretion. Under basal conditions, the plasma glucocorticoid concentrations show robust circadian (24 h) fluctuations with a peak around the sleep/wake transition. In humans, plasma cortisol increases in early morning and reaches a trough at midnight [reviewed by [71]], while in nocturnal rodents, corticosterone shows an inverted phase, which is correlated with nocturnal activity [reviewed by [72]].

This circadian rhythm in glucocorticoid secretion is controlled at different levels and by multiple pathways. The PVN receives direct synaptic input from the SCN and pocesses a self-sustained molecular clock [73]. In addition to the parvocellular endocrine neurons, the preautonomous PVN neurons contribute to rhythmic glucocorticoid secretion by rhythmic activation of the adrenal cortex via the sympathetic nervous system [74]. The adrenal cortex itself gates its response to ACTH via an intrinsic circadian clock that regulates sensitivity of the adrenal cells to ACTH stimulation [reviewed by [72]]. Rhythms in glucocorticoids control subsidiary circadian oscillators via binding to glucocorticoid receptors (GR) and mineralocorticoid receptors (MR) [71,75]. Moreover, the transcriptional activity of GR is controlled by the molecular clockwork [76]. Physiological levels of glucocorticoids exert a beneficial effect on NPCs, while elevated levels, due to chronic stress or pharmacological application, impair NPC proliferation via cyclin-dependent kinase 5-mediated suppression of neurotrophic factors such as brain-derived neurotrophic factor (BDNF) and enhances NPC apoptosis in the SGZ [77,78]. Rhythms in corticosterone levels are essential for rhythmic variation in the number of NPCs in the SGZ [36]. Similarly, disruption of glucocorticoid rhythms leads to long-lasting changes in dendritic tree complexity and decreased spine density of newborn hippocampal neurons, especially thin and stubby spines [79]. Thus, glucocorticoids modulate rhythms in proliferation and differentiation of NPCs.

Consistently, oscillation of glucocorticoid levels is crucial for the buffering of anxiety [80], emotional memory [81] and executive function [82], while flattened cortisol slope is associated with impaired cognitive performance and memory deficits [83,84]

Glucocorticoids are also implicated in aging of the neurogenic niches. Recently, NPCs were classified according to GR expression into a GR-negative^(−)^ subpopulation that rapidly depletes by middle age, and a GR-positive^(+)^ subpopulation that loses its proliferative capacity linearly with advancing age. Furthermore, rhythms in glucocorticoids preserve a quiescent NPC pool in the aging hippocampus via regulation of cell cycle progression and DNA methylation and thus, contribute to a neuroplasticity reserve in the aging brain [79]. Thus, rhythms in glucocorticoids modulate age-dependent changes in adult neurogenesis.

#### 3.2.2. Melatonin

Melatonin (N-acetyl-5-methoxytryptamine) is rhythmically synthesized by the pineal gland. Rhythms in pineal melatonin secretion are controlled by the SCN through the sympathetic nervous system and rhythmic expression of the rate limiting enzyme arylalkylamine N-acetyltransferase [reviewed by [85]]. In clinical studies, melatonin and its metabolites are reliable markers for the integrity of the circadian system [reviewed by [86]]. However, many laboratory mouse strains, including the most commonly used C57BL/6, are melatonin-deficient [87]. In both nocturnal and diurnal animals including humans, serum melatonin levels are high during the night and low during the day. It serves as an important signal within the brain and the body through melatonin receptors type 1 and type 2 (Mt1, Mt2) [reviewed by [88,89]], encoding the phase and the duration of the night. Melatonin acting on Mt1/Mt2 receptors, which are distributed throughout the hippocampus including the SGZ, has modulatory effects on neurogenesis [reviewed by [90]]. Specifically, only melatonin-proficient mice with functional Mt1/Mt2 receptors show a time-of-day-dependent fluctuation in the number of proliferating NPCs and in antiphase of apoptotic cells within the hippocampal neurogenic niche [91].

In pinealectomized rats [92], and in the offspring of pinealectomized mothers [93], adult hippocampal neurogenesis was reduced but was rescued by melatonin treatment. Thus, melatonin, which can pass the placenta barrier, primes adult neurogenesis in the offspring [93]. Melatonin modulates NPC proliferation, the number of DCX+ neuronal precursor cells and improves survival as well as dendritic maturation of the newborn neurons in the hippocampus [94,95]. In addition, melatonin potentiates the stimulatory effect of wheel running on neurogenesis [96] and opposes the deleterious effect of aging [97]. However, the underlying mechanisms remain largely unclear. However, it is suggested that melatonin modulates mitochondrial DNA copy number and oxidative phosphorylation proteins including COX I, COX IV, ATP-5b and NDUFB8 [98]. Interestingly, melatonin is able to counteract the neurotoxic effects of reactive oxygen species (ROS) (see below). Recently, it was shown that melatonin enhances hippocampal adult neurogenesis by preventing intracellular oxidative stress and increasing antioxidant activity [99]. Moreover, melatonin induces a neuroprotective effect in sleep deprivation and modulates expression of the neurotrophic factor insulin-like growth factor 1 (IGF-1) [reviewed by [100].

### 3.3. Neurotrophic Factors

Adult neurogenesis is regulated by a variety of neurotrophic factors including BDNF, vascular endothelial growth factor (VEGF), and IGF-1 [101]. Some effects on neurotrophic factors by systemic and cellular factors under the control of the circadian system have been discussed already. Here, we will discuss the circadian regulation of neurotrophic factors in more detail. BDNF is highly expressed in the brain and is involved in regulation of synaptic plasticity [102] and various steps of adult neurogenesis [101]. In rodents, mRNAs of BDNF and its receptor trkB as well as BDNF protein levels show significant time-of-day-dependent oscillations within the hippocampus, other parts of the cerebral cortex and the SCN [103,104]. Furthermore, in humans, plasma BDNF level show time-of-day-dependent fluctuation [105,106]. This suggests that BDNF might be involved in regulation of rhythms of several brain functions including adult neurogenesis. The time-of-day-dependent rhythms of BDNF and cortisol are correlated [107] and are suggested to be mediated via CREB-mediated pathways [108]. Importantly, our own studies show that CREB plays a significant functional role in entrainment of the SCN [109] and light-induced clock gene expression in the SCN [110]. Thus, SCN molecular clockwork entrainment and control of BDNF expression share common signal transduction pathways. Interestingly, BDNF transcripts in SCN varied significantly when hamsters were housed under a long or short photoperiod, indicating light regulation of BDNF level [111]. In the hippocampus, the level of BDNF protein decreased under constant conditions [104]. However, little is known if the rhythm of BDNF persists under constant environmental conditions and thus under intrinsic circadian control or if it is dependent on the light/dark cycle.

IGF-1, which is primarily produced by the liver, also enhances adult neurogenesis. Reduced IGF-1 levels contribute to dementia and age-related cognitive impairments, while higher levels may counteract neurodegeneration [112]. Plasma IGF-1 levels reveal a pronounced 24 h rhythm in rats with a peak level at the end of the dark phase and beginning of the light phase [113]. Inflammatory conditions could impair the IGF-1 rhythms [113], while improved circadian rhythm sleep–wake disorder is associated with increase in IGF-1 level [114]. Interestingly, melatonin is implicated in IGF-1 regulation via multiple pathways involved in the energy metabolism, e.g., PI3K–Akt signaling and glycogen synthase kinase 3 β (GSK3β) [reviewed in [100]]. In addition, there is an inverse relationship between IGF-1/IGF-binding proteins (IGFBPs) and cortisol level. These data point to a regulatory role of the circadian system on IGF-1 levels, probably via hormone-dependent pathways.

VEGF is produced mainly by platelets and leucocytes in the plasma [115] and by endothelium in multiple organs including the brain [116]. VEGF represents a crucial regulator of angiogenesis, endothelial functions as well as retinal development and is essential for adult neurogenesis and neuroprotection [117]. VEGF seems to be directly regulated by the molecular clockwork as Bmal1 directly targets the *Vegf* gene promoter leading to enhanced expression of VEGF in zebra fish [118]. In humans, VEGF is increased during the postprandial phase, associated with increased glucose level [119]. Taken all together, the rhythms in neurotrophic factors are driven by the circadian system either directly downstream of the molecular clockwork or indirectly via light/dark cycles, rhythmic hormones, e.g., cortisol and melatonin, as well as rhythmic behavior, e.g., feeding/fasting or locomotor activity.

### 3.4. Neurotransmitters

Signaling of neurotransmitters within the brain, including acetylcholine, noradrenaline (NE), dopamine, GABA, serotonin and glutamate is implemented not only in neuronal function but also in the formation of newborn neurons; however, this is regulated differentially at the neurogenic niches [120]. Several brain regions, including the hippocampus show daily rhythms in various neurotransmitters. For instance, acetylcholine shows periodic release in the dorsal hippocampus with suppressed levels during the light phase, followed by rapidly increased release after the onset of the dark phase [121]. Additionally, a circadian rhythm was observed in the level of the NE metabolite, 3-methoxy-4-hydroxyphenylglycol, in the hippocampus [122]. GABA release in the cerebral cortex also shows time-of-day-dependent variation being higher during the night in hamsters [123]. Serotonin receptor signaling shows a daily rhythm in the hippocampus, which is anti-phasic to plasma corticosterone levels, but independent on stress [124]. The molecular clockwork controls rhythmic midbrain dopaminergic activity [reviewed by [125]] and dopamine receptor signaling in the hippocampus [126]. D2 and D3 dopamine receptors are expressed in SVZ-derived NPCs and dopamine receptor activation promotes adult neurogenesis in an acute Parkinson’s model [127]. These data suggest an interaction of the molecular clockwork and dopamine-mediated enhancement of adult neurogenesis at least under pathological conditions. Moreover, mice with impaired molecular clockwork function show a dysregulation of neurotransmitter balance as well as structural and functional deficits [128,129].

Taken all together, further studies are needed for better understanding of the control of rhythmic neurotransmitter signaling in the neurogenic niches by the different components of the circadian system and by the molecular clockwork under physiological and pathological conditions.

### 3.5. Behavior and Physiology

#### 3.5.1. Sleep

Sleep/wake phases result from reciprocal excitatory and inhibitory circuits that lead to consciousness and sleep states, respectively. The preoptic hypothalamic area (POHA) plays a crucial role in sleep–wake regulation. It includes sleep-active neurons located in the ventrolateral preoptic area (VLPO) and the median preoptic nucleus (MnPO) that release GABA and thus inhibit the wake-promoting system. The wake-promoting system includes orexinergic neurons in the lateral hypothalamus and histaminergic neurons in the tuberomammillary nucleus in addition to the reticular activating system in the brain stem [reviewed by [130]].

In mammals, sleep consists of cycles of rapid eye movement (REM) sleep and different stages of non-REM (NREM) sleep. In each stage, there are synchronized neuronal activities that are essential for brain functions including memory consolidation [131], probably due to promoting synaptic strength [132].

Circadian regulation of the sleep/wake cycle represents the most pronounced 24 h pattern in our behavior. The circadian system is essential for alignment of sleep/wake cycles to the 24 h day and for sleep quality. Adequate sleep duration and quality is crucial for physical and mental health. Inadequate sleep deteriorates several brain functions [reviewed by [133,134]]. Sleep deprivation, sleep restriction, sleep fragmentation, or REM-specific sleep deprivation could impact adult neurogenesis and, consequently, negatively impact the learning ability via multiple routes. Sleep deprivation targets a wide range of epigenetic modifications of gene expression in the hippocampus, importantly, the BDNF signaling pathway [135]. Importantly, sleep deprivation leads to an increase in levels of glucocorticoids, which interfere, as mentioned above, with circadian rhythms. However, sleep deprivation in adrenalectomized rats maintained on replacement corticosterone also showed impaired adult hippocampal neurogenesis [136], suggesting a direct effect of sleep loss independent of stress response.

The adult-born neurons show overall reduced neuronal activity; however, they show reactivation specifically during REM sleep. This scarce activity is essential for REM sleep-dependent memory consolidation [137,138], indicating that sleep is essential for newborn neuron functionality. Interestingly, mice lacking adult neurogenesis reveal shorter sleep time due to NREM sleep disruption and higher sleep fragmentation [139], suggesting a bidirectional interaction between sleep and adult neurogenesis. Importantly, exogenous administration of melatonin, inducing alignment of circadian rhythms [reviewed by [140]], increases NPC proliferation in sleep-deprived mice and provides a neuroprotective effect against the deleterious effects of REM sleep deprivation [141]. In particular, the neurogenic niche within the third ventricle (discussed below) contributes to the addition of newborn neurons to the sleep-promoting system in the POAH and suppression of NPC proliferation and differentiation within this niche induces ageing-like disturbance of the sleep–wake architecture [142]. These data indicate that there is interlacing regulation of adult neurogenesis by sleep and the circadian system. Thus, optimization of synchronization within the circadian system may rescue impaired adult neurogenesis under physiological and pathological conditions.

#### 3.5.2. Feeding–Fasting Cycles

Feeding–fasting cycles coincide with sleep–wake cycles controlled by the circadian system and entrained to rhythmic environmental factors [reviewed by [143]]. These defined feeding–fasting patterns enhance daily rhythms in metabolic state, digestion, immunity and several brain functions. In humans, dysregulation of the circadian system due to sleep disruption, jet-lag or shift work alters the feeding–fasting pattern and allows for prolonged evening eating and increases caloric intake [reviewed by [144]]. Time-restricted feeding/fasting, in which food intake is restricted to a specific time of the day and daily fasting period > 12 h, is considered to induce beneficial effects in decreasing blood cholesterol levels, body weight and inflammation [143]. Periodic diet mimicking fasting, without reduction in total caloric intake, has also been shown to promote adult neurogenesis and improve hippocampal-dependent cognitive performance in mice. These positive effects were correlated with alterations in systemic factors, including IGF-1 levels and receptor expression as well as PKA/CREB-dependent regulation of NeuroD1 [145]. Furthermore, dietary restriction increases the BDNF level in the hippocampus [146]. Consistently, a recent study demonstrated a positive association between time restricted feeding and the cognitive status in humans [147]. These data indicate that definitive rhythmic pattern in feeding–fasting, even without restriction of total caloric consumption, positively regulates adult neurogenesis and hippocampal dependent learning and memory. Thus, the circadian system might also impact adult neurogenesis through regulation of feeding–fasting timing, which in turn could be manipulated and utilized as a non-invasive novel therapeutic strategy for cognitive decline.

#### 3.5.3. Locomotor Activity

The circadian system determines rhythms of locomotor activity [148] via various outputs including vasopressin signaling [149]. There is a large body of evidence suggesting that physical activity and running increase adult neurogenesis and improve cognitive abilities via multiple neuronal and endocrine mediators including serotonin, BDNF, VEGF, IGF-1, platelet growth factor, fibroblast growth factor (FGF-2), NeuroD1 and endorphins [150] [reviewed by [151]].

Amount and frequency of physical activity positively correlate with cognition and mental health in humans [152] and in rodents [reviewed by [153]] and even short periods of physical activity are able to trigger neurogenic processes [150].

Interestingly, the circadian phase of physical activity also plays a crucial role in modulation of neurogenesis, as neurogenesis was enhanced when mice had scheduled access to a running wheel during the middle of a dark period, but not at a light or the beginning of a light period [154]. This suggests that the circadian system may indirectly modulate adult neurogenesis via regulation of rhythms in locomotor activity.

#### 3.5.4. Body Temperature

Endogenously generated daily rhythms of body temperature are confirmed in many mammalian species. Moreover, the stability of its waveform and its range of oscillation across the day is strongly correlated to the robustness of the circadian system [155,156]. Although the peak in body temperature rhythm is associated with the phase of activity, robust rhythmicity in body temperature is not only due to rhythmic locomotor activity, but generated autonomously by a neuronal network regulating the temporal balance between heat production and heat loss under the control of the circadian system [155]. Interestingly, our own data suggest that this regulation involves heat shock factor- 1 (HSF1), as HSF1-deficient mice show increased core body temperature (hyperthermia) while overall locomotor activity is decreased [157].

Scarce data are available regarding the role of body temperature rhythms in adult neurogenesis. Indeed, contrasting findings demonstrated the effect of hypothermia, as a line of treatment for brain hypoxia, on adult neurogenesis. Mild hypothermia remarkably promoted the number of Bromodeoxyuridine-positive (BrdU+) and DCX+ cells in the rat hippocampus after traumatic brain injury, supporting the idea that cooling inhibits multiple cell injury pathways [158]. However, this beneficial effect might be limited to post insult models, as hypothermia appears to have detrimental effects on neurogenesis in the neonatal healthy rat [159]. An underlying mechanism is suggested to be altered levels of hormones and neurotrophic factors due to hypothermia, including glucocorticoids.

On the other hand, hyperthermia seems to induce neurogenesis. A study by Tao et al. showed that febrile convulsions in young mice could enhance structural plasticity and hippocampal-dependent learning and memory [160], consistent with clinical observations from children who experienced a febrile seizure and performed better in cognitive tasks than the age-matched controls [161]. In addition, external temperature can also impact adult neurogenesis. Exposure to a mild heated environment for a long period (28 days) promotes post-traumatic adult neurogenesis in SVZ and the hippocampus. This neuroprotective effect was mediated by angiotensin ll type 2 receptor (AT2) and BDNF signaling [162]. Similarly, short-term exposure for 1h/7days was also capable of increasing the number of DCX+ cells in the rat hippocampus and *Vefg* mRNA expression in hippocampal astrocytes that was mediated via angiotensin II type 1 receptor (AT1) [163].

Indeed, a potential regulatory role of body temperature on adult neurogenesis may fit with rhythms in NPCs proliferation (discussed in next chapter) that peak during the higher body temperature/active phase and show a trough level during lower body temperature/rest phase. However, further research is needed to confirm this correlation.

### 3.6. Redox State

ROS are formed during metabolic processes or during responses to stress exposure. ROS encompass a diversity of radicals including: singlet oxygen, superoxide anion radical, hydroxyl radical (OH) and hydrogen peroxide (H_2_O_2_). Although mild peroxidation can have beneficial effects, higher levels of ROS have cytotoxic effects, especially by interfering with biomolecules including fatty acids in cell/organelle membranes. Furthermore, DNA, especially in mitochondria, is vulnerable to ROS damage leading to mutations, carcinogenesis or cell death. Thus, reduction-oxidation (redox) balance is fundamental for health [reviewed by [164]]. Elevated ROS levels have detrimental effect on adult neurogenesis and results in insufficient brain repair and progressive neurodegeneration [165]. Mice lacking ROS buffering due to superoxide dismutases (SODs) deletion show significant reduction in newborn neurons [reviewed by [166]]. Nevertheless, ROS appear to be a double-edged sword depending on duration of exposure and proliferation/differentiation stage of stem/progenitor cells. The maintenance of quiescent neural stem cells require reduced ROS levels; however, the proliferating NPCs produce transiently high endogenous ROS levels that significantly alter their self-renewal capacity and adult neurogenesis in SVZ, probably via AKT-mediated signaling [167]. High ROS levels shift the differentiation potential of newborn cells in the hippocampus to the astrocytic lineage [168].

Circadian rhythms of ROS production and elimination have been shown in various tissues [169,170]. In rodent SCN slices, an endogenous oscillation of redox state, with a remarkable oxidized state in the early night time and a reduced state during the day time, have been shown to match the rhythms of neuronal hyperpolarization and depolarization, respectively [171]. Importantly, this redox state fluctuation requires functional molecular clockwork [171]. In Bmal1-deficient mice, ROS levels are high in various tissues including the brain, which is associated with accelerated aging [172,173] and impairment of tissue/organ and brain function [174]. We could also show that adult neurogenesis in Bmal1-deficient mice is affected, at least in part, as a consequence of high ROS levels [32,33].

### 3.7. Clock Genes/Molecular Clockwork

In the neurogenic niches, NPC proliferation, differentiation and survival are affected in mice with mutations/deletions in clock genes indicating a functional link between the molecular clockwork and adult neurogenesis. In order to distinguish the role of the molecular clockwork at the cellular level from the systemic effects of a disrupted circadian system due to clock gene deletions/mutations, in vitro studies are mandatory. In isolated hippocampal NPCs from mice with clock gene deletions, proliferation, differentiation and survival are affected, indicating a role of clock genes on all levels of adult neurogenesis at the cellular level [38].

#### 3.7.1. Proliferation and Apoptosis

Generally, molecular clockwork has been implicated in the cell proliferation in various organs via multiple cell cycle modulators, e.g., the oncogene cMyc, the tumor suppressor genes p21 and p53, wee1 (G2/M checkpoint regulator), ATM/CHK1 (DNA repair), which are regulated through molecular clockwork [175]. Moreover, clock proteins modulate the DNA damage by interacting with Timeless in a time-of-day-dependent manner in fibroblasts [176]. There is also increasing evidence for the presence of functional molecular clockwork in NPCs and its important role in adult neurogenesis. In the SGZ, clock genes and NPC proliferation shows daily rhythms which persist in constant darkness, thus circadian [177]. Hippocampal NPC proliferation fluctuates across the light/dark phases in hamsters [178] and mice, with higher proliferation levels in the night/active phase than in the day/inactive phase under a standard photoperiod [179], and constant darkness [177]. These rhythms are abolished in mice with mutations/deletions of *Bmal1, Per2* [177], or *Rev-erbα* [179], thus dependent on a functional molecular clockwork.

BrdU can be used to label solely the progenitors entering S-phase, if the animals are sacrificed shortly after BrdU application, before the cell cycle proceeds and another round of cell division takes place [180]. On the other hand, the phospho-histone H3 (PH3) is used as a marker of M-phase cells. These two markers make it possible to distinguish between NPCs in different stages of the cell cycle. It has been reported that the number of progenitors entering S-phase do not fluctuate across the day [181], while the number of progenitors in the M-phase showed a significant increase during the dark/activity phase. These observations were limited to SGZ, whereas, no change was found in the other neurogenic niche of the SVZ throughout the day, indicating a region-dependent phenomenon [181]. Matsumoto et al. described a coincidence between the proliferation rhythms and expression of cyclin proteins, which are essential for progression through the cell cycle. This supports the hypothesis that hippocampal NPCs enter the cell cycle regardless the time of day. During the day/rest time, their progression into M-phase is inhibited, presumably due to G2 arrest. At nighttime, the NPCs proceed to M-phase, resulting in the production of more newborn cells [182,183].

The molecular clockwork is critical for a proper control of NPC proliferation. *Rev-erbα* knockout mice show an enhanced cell-division of hippocampal NPCs [179]. In glioblastoma cell culture, proliferation is suppressed by REV-ERBα and its target fatty acid binding protein 7 (FABP7), which is a marker for NPCs [184]. Similarly, mice with a *Per2* mutation show an increase in the number of NPCs and immature newborn neurons [185]. Consistently, PER2 overexpression could suppress proliferation in glioma stem cells via the Wnt/β-catenin signaling pathway [186]. Interestingly, SOX2, which is essential for proliferation of neural stem cells, promotes the activation of the *Per2* promoter [187].

A study by Bouchard-Cannon has shown that the absence of PER2 abolishes the gating of cell-cycle entrance of quiescent neuronal precursors, whereas Bmal1-deficiency results in constitutively high levels of proliferation and delayed cell-cycle exit [177]. Mathematical models suggest a clock-driven expression of a cell-cycle inhibitor that targets the cyclin D/Cdk4-6 complex [177].

Consistent with ROS-dependent accelerated aging in Bmal1^−/−^ mice (see above), adult neurogenesis shows a strong age-dependent alteration. While juvenile Bmal1^−/−^ mice exhibited enhanced NPCs proliferation [177], it is unchanged in 8-week-old young adults [188] and reduced in 4-month-old adults [32]. Premature aging of the hippocampal neurogenic niche in Bmal1^−/−^ mice is presumably a consequence of age-dependent upregulation of the cell cycle inhibitor p21(Waf1/CIP1), reduced expression of *Bdnf*, and increased oxidative stress [32]. However, the role of Bmal1 in NPCs proliferation appears to be region-specific, as the proliferation of NPC is also reduced in the proximal limb of the RMS but not in the SVZ or the distal RMS [33].

Interestingly, the increase in NPC proliferation in *Per2* mutant mice is associated with an increase in cell death [185], while the reduced proliferation in adult Bmal1^−/−^ mice is associated with enhanced survival [32], suggesting intact compensatory mechanisms despite the absence of essential clock genes. Even in young adult Bmal1^−/−^ mice, where NPC proliferation is not affected (see above), survival of newborn cells is enhanced and cell death is reduced, [188], suggesting a role of Bmal1 in the pruning of newborn cells.

In vitro studies with neurospheres from mice with deletions of essential clock genes such as *Bmal1* and *Cry1/Cry2* confirm that clock genes control NPC proliferation at the cellular level [38]. However, in both SVZ-derived [189] and hippocampus-derived neurospheres [38], rhythmic expression of clock genes and of clock-related genes only starts during differentiation into neural progenitor cells. Thus, during the stem cell state, clock genes control proliferation despite a not yet functional molecular clockwork [190].

#### 3.7.2. Differentiation and Migration

Neuronal differentiation seems to undergo daily rhythms, supported by the observation that the number of BrdU+ cells co-expressing the early neuronal marker DCX during night time is twice as high as during day time [183]. A computational model for neural fate decisions involving the clock-related *cis* element via regulation of Notch signaling pathway has been suggested by Wang and colleagues and provides a candidate mechanism for regulation of neuronal differentiation by the molecular clockwork [191]. In Bmal1^−/−^ mice, hippocampal NPCs show an increased differentiation into the astroglial lineage at the expense of the neuronal lineage, presumably as a consequence of reduced *Bdnf* expression and increased oxidative stress [32].

In neurospheres, silencing of *Clock* or *Bmal1* decreases the percentages neuronal precursor cells and the expression levels of NeuroD1, which is implicated in NPC differentiation [189]. Similarly, NPCs from Bmal1^−/−^ mice show a higher differentiation into glia rather than neurons in vitro [38], indicating a role of Bmal1 in NPC differentiation at the cellular level.

Although time-of-day-dependent rhythms in migration/mobilization of hematopoietic stem cells have been described [192], to our knowledge it is not known if rhythms in migration apply also to NPCs. In glioblastoma cell culture, migration is suppressed by REV-ERBα and its target FABP7, termed brain lipid-binding protein, which is implicated in glial and neuronal differentiation in primary cell cultures as well as in migration of immature neurons during embryonic development of the cerebral cortex [193]. Clock regulates expression of genes involved in neural migration including *Prdx3*, which is involved in cellular redox state regulation, and, consequently, knockdown of clock in neurospheres results in increased migration distance [194]. Similarly, Bmal1^−/−^ derived NPCs migrate in vitro for a longer distance and at a higher velocity, presumably as a consequence of dysregulated ROS detoxification, increased levels of the ROS-sensitive mediator of actin polymerization p-cofilin and more pronounced filopodia formation [33], consistent with an increased migration of newborn neurons in both neurogenic niches [32,33].

#### 3.7.3. Neurogenesis-Related Brain Function

Mice with deletions/mutations of clock genes show changes in brain function in general [reviewed by [195]] and of neurogenesis-related brain functions such as hippocampus-dependent learning and memory [173,196,197,198] as well as olfactory function [199]. The dysregulated adult neurogenesis in the SGZ of adult (3-4 month old) *Bmal1*-deficient mice [32] is associated with impaired hippocampus-dependent cognitive performance [173], before the pathological changes in the brain [174] occur. However, although Per2 seems to play an important role in adult neurogenesis (see above), hippocampus-dependent learning is not affected in *Per1/Per2* mutants [200,201]. In forebrain-specific *Bmal1*-deficient mice hippocampus-dependent learning and memory is impaired [202] while major olfactory function and adult neurogenesis in both neurogenic niches are intact [203], indicating a role of Bmal1 in hippocampal function in addition to its role in adult neurogenesis. Odor discrimination largely depends on integrated interneurons in the olfactory bulb [49]. The overall odor discrimination sensitivity is intact *Bmal1*-deficient mice; however, the circadian oscillation of discrimination sensitivity is ameliorated [199]. Nevertheless, further studies are required to elucidate the effect of the circadian system on the function of newborn neurons in the olfactory bulb.

#### 3.7.4. Hypothalamic Neurogenic Niche

In addition to the “classical” neurogenic niches reviewed above, there is increasing evidence for important neurogenic zones in the hypothalamus, which contribute to hypo-thalamic functions [204] such as energy metabolism [205] and sleep–wake regulation [130,142]. In the hypothalamic parenchyma, scattered neurons express Sox2, a selective marker for neural stem cells [reviewed by [41]]. In addition, two neurogenic zones in the third ventricle, which are surrounded by the hypothalamus, can be distinguished [206]. One is located in the lateral walls at the level of the PVN and the arcuate nucleus (hypothalamic ventricular zone, HVZ), the other is the hypothalamic proliferating zone (HPZ) formed by specialized ependymal cells, tanycytes, at the bottom of the third ventricle in the median eminence [41]. Tanycytes have a radial-glia-like morphology and express several markers typical for NPCs, including nestin, vimentin and doublecortin-like protein [207,208] and Sox2 [41,209,210]. Four types of radial-glia-like tanycytes can be distinguished according to their gene profile, morphology, location and function [41]. Of note, the processes of β-tanycytes are in contact with terminals of GnRH-expressing neurons and the endothelial cells of the hypothalamo-pituitary portal system, which are modulated by hypothalamic T3 implicated in seasonal rhythms [reviewed by [211]]. The NPCs in HVZ migrate into the surrounding hypothalamus including the POHA, SCN and arcuate nucleus where they differentiate into neurons, astrocytes or oligodendrocytes [reviewed in [130]]. The role of the hypothalamic neurogenic zone for adult neurogenesis remains so far controversial. Like in the “classical” neurogenic niches, the proliferation in the hypothalamic neurogenic zones, especially of β-tanycytes, is substantially higher in juvenile than in adult animals [209,210]. In the adult hypothalamus, GFAP-positive dorsal α-tanycytes constitutively give rise to new tanycytes, astrocytes and a sparse number of neurons [212]. Moreover, they form neurospheres and keep their self-renewing capacity in vitro in contrast to β-tanycytes and parenchymal Sox2 expressing cells [212]. Interestingly, also in the SCN, Sox2 and additional markers for NPC and neural progenitor cells are expressed including Sox11, Zfhx3, Btg1, Nr2f2, Rora, Rorb [187,213] and DCX [209]; although there is a lack of obvious neurogenesis in this brain region. The exact function of these genes in SCN cells is not clear yet; however, Sox2-dependent gene expression in the SCN promotes the robustness of circadian rhythms [187,213]. The adult neurogenesis within the hypothalamic neurogenic niche seems to be regulated by common factors similar to those within the ‘’classical niches’’ [reviewed in [130]], especially FGF-signaling, which governs α-tanycyte [212] proliferation. However, intrinsic and extrinsic regulatory factors that specifically affect the hypothalamic neurogenic niche and their interaction with the circadian system still need further elucidation.

## 4. Conclusions

**Figure 3:**
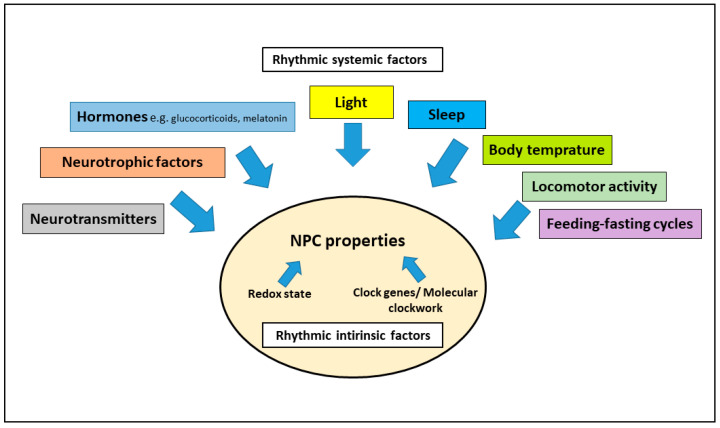
Modulation of adult neurogenesis/NPC properties by the circadian system via rhythmic systemic factors and via rhythmic intrinsic factors.

There is increasing evidence that the circadian system modulates the multistep process of adult neurogenesis via rhythmic systemic factors or via NPCs’ intrinsic factors, such as the redox state and clock genes/molecular clockwork (Figure 3). Better understanding of how the circadian system modulates adult neurogenesis could help develop new therapeutic approaches to improve mood-related and cognitive impairments associated with chronodisruption induced by aversive light regimes or neuropsychiatric and neurodegenerative diseases.
